# Metastatic Merkel cell carcinoma and myasthenia gravis: contraindication for therapy with immune checkpoint inhibitors?

**DOI:** 10.1186/s40425-019-0626-9

**Published:** 2019-05-29

**Authors:** Anne Zaremba, Eleftheria Chorti, Finja Jockenhöfer, Saskia Bolz, Selma Sirin, Martin Glas, Jürgen C. Becker, Selma Ugurel, Alexander Roesch, Dirk Schadendorf, Elisabeth Livingstone, Tim Hagenacker, Lisa Zimmer

**Affiliations:** 1 Department of Dermatology, University Hospital Essen, Essen, Germany; 2 Department of Neurology, University Hospital Essen, Essen, Germany; 3 Department of Diagnostic and Interventional Radiology and Neuroradiology, University Hospital Essen, Essen, Germany; 4 Division of Clinical Neurooncology, Department of Neurology, University Hospital Essen, Essen, Germany; 5 Translational Skin Cancer Research, German Cancer Consortium (DKTK), Partner Site Essen/Düsseldorf, Essen, Germany

**Keywords:** Merkel cell carcinoma, Myasthenia gravis, Immune checkpoint inhibitor, Adverse events, Immunotherapy

## Abstract

**Background:**

PD-1/PD-L1 inhibitors are promising approaches for advanced Merkel cell carcinoma (MCC). Nevertheless, these inhibitors bear a high risk for induction of immune-related adverse events (irAEs), particularly flares of preexisting autoimmune diseases. Neurological irAEs of PD-1/PD-L1 inhibitors are possibly underestimated and potentially fatal toxicities. Additionally, exacerbations of preexisting myasthenia gravis (MG) with a high MG-specific-related mortality have been reported.

**Case presentation:**

A 61-year-old woman with a history of MG since 2005 was treated with azathioprine and pyridostigmine after thymectomy. In March 2016, she was diagnosed with MCC. Six months later the tumor had progressed to stage IV and metastases were detected in lymph nodes and the pancreas. The immunosuppressive therapy was therefore changed to mycophenolatmofetil (MMF) and an immune checkpoint blockade with the PD-1 inhibitor pembrolizumab was initiated in November 2016. Due to MMF-induced liver toxicity, MMF was switched to cyclosporine A (CsA) with normalized liver transaminases six weeks later. After six cycles of pembrolizumab the patient achieved a partial response. Follow up analysis sixty-five weeks later revealed a long-lasting tumor response with a partial remission of pancreatic and inguinal metastases and no flare of MG.

**Conclusions:**

Patients with a preexisting MG can be considered for treatment with immune checkpoint inhibitors if they have a life-threatening cancer and if other effective, long-lasting treatment options are not available. The risks and benefits of therapy should be weighed in a multidisciplinary setting and should be discussed thoroughly with the patient. Exacerbation of underlying MG can be potentially life-threatening and requires close monitoring in collaboration with neuromuscular specialists.

## Background

Blocking antibodies for programmed cell death protein 1 (PD-1) are commonly used for the treatment of metastatic melanoma and other tumours[1–3]. Although advanced Merkel cell carcinoma (MCC) responds to chemotherapy, responses are seldom durable, showing a median progression-free survival of only 94 days [4]. As MCC cells often express programmed cell death protein ligand 1 (PD-L1) and Merkel cell polyomavirus (MCPyV)-specific T cells express corresponding PD-1, blockage of the PD-1 immune inhibitor pathway is of interest and PD-1/PD-L1 inhibitors have been shown to be a promising approach for the treatment of advanced MCC [5, 6]. Recently, three phase II open-label clinical trials of the PD-1/PD-L1 inhibitors pembrolizumab, nivolumab and avelumab in patients with metastatic MCC have demonstrated high and durable response rates of 57, 73 and 62.5%, respectively [5–7]. Nevertheless, PD-1/PD-L1 inhibitors also bear the risk for inducing immune-related adverse events (irAEs). The most frequent irAEs are skin toxicities, colitis, hepatitis and endocrinopathies [1]. Rare irAE include pneumonitis, nephritis, neurological and cardiological side-effects. Neurologic irAEs of the central and peripheral nervous system (PNS) have been reported in up to 12% of patients treated with immune checkpoint inhibitors [8–10]. Common neurologic irAEs of the PNS include mild to moderate peripheral neuropathies, but cases of life-threatening and fatal cases of Guillain–Barré syndrome, necrotizing myositis and myasthenic syndromes have been reported [7, 8]. In the literature, 23 cases of MG after immunotherapy with checkpoint inhibitors have been described, the majority being de novo cases (72.7%), but also some cases of exacerbations of a preexisting MG (18.2%) or subclinical MG (9.1%) [1]. MG-related mortality was estimated at 30.4% [1]. Only limited experience exists regarding therapy with immune-checkpoint inhibitors in patients with preexisting autoimmune disorders, as they are often excluded from clinical trials [11].

In this case report, we describe our recent experience with administration of pembrolizumab in a patient with metastatic MCC and well-controlled MG on immunosuppressive therapy.

## Case presentation

A 61-year-old woman was diagnosed with anti-acetylcholine receptor antibody (ACh-R) positive MG in 2005. Initially, only ocular signs were present, but systemic symptoms arose over time showing a relapsing course. During her last myasthenic crisis in 2009 a thymectomy was performed and an immunosuppressive therapy with azathioprine in combination with pyridostigmine was initiated. Neurological symptoms were fully controlled without residual symptoms. Doses of azathioprine and pyridostigmine remained stable during the regular three-monthly neurologic screening visits. In March 2016 a MCPyV-positive MCC measuring > 5 cm in diameter with a tumor thickness of 22 mm was detected on her right gluteal side. After wide local excision of the primary tumor with a 3 cm safety margin and a negative sentinel lymph node biopsy of the right groin, she received an adjuvant radiotherapy of the primary tumor site. The patient underwent a rigorous follow-up scheme with clinical examinations and ultrasound of the regional lymph nodes every six weeks and yearly chest X-ray and abdominal ultrasound were planned. In September 2016, six months after the initial diagnosis of MCC, ultrasound of the right inguinal groin showed enlarged lymph nodes. A subsequent positron emission tomography (PET)-computed tomography (CT) confirmed right inguinal lymph node metastases. Additionally, metastases of the pancreatic tail and its surrounding lymph nodes were detected. To exclude a secondary malignancy, a biopsy from the pancreas was performed confirming MCC metastasis. Due to the extensive metastatic spread of the MCC, immune-checkpoint therapy with a PD-1 inhibitor was recommended by our interdisciplinary tumor board. The risks (i.e. exacerbation of preexisting MG with potential lethal outcome) and benefits (i.e. life-threatening metastatic MCC with a response rate of around 60% to PD-1/PD-L1 inhibitors) of PD-1/PD-L1 inhibitor therapy was discussed thoroughly with our neuromuscular specialists and the patient. As treatment with azathioprine has been identified as a risk factor for the development of non-melanoma skin cancer in transplant recipients and myasthenia patients [12–15] therapy for MG was switched from azathioprine to mycophenolatmofetil (MMF) (500 mg 1–0-1). After extensive education of the patient and the patients’ family, immunotherapy using PD-1 inhibitor pembrolizumab at a dose of 2 mg/kg every three weeks was started in November 2016. At the time of therapy initiation avelumab was neither approved for MCC nor available to our skin cancer unit [7]. Therefore, due to published overall response data at that time [6], pembrolizumab was chosen.

**Fig. 1:**
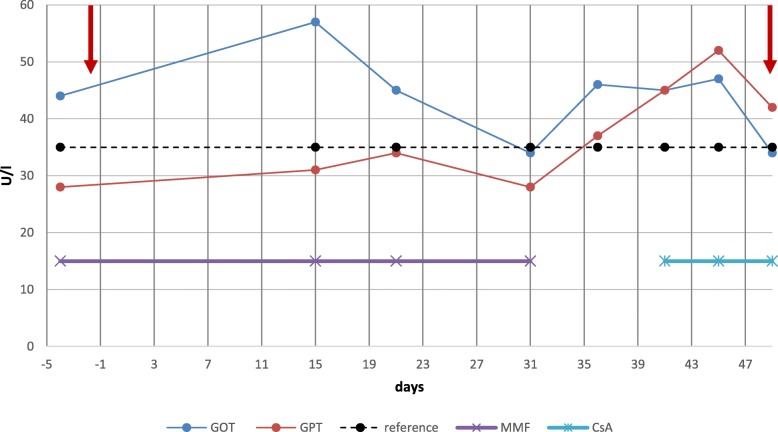
Course of liver enzymes GPT (ALT) and GOT (AST) over time. After the first course of pembrolizumab (day 0), a grade 1 transaminitis (CTCAE; version 4.0) reaching values up to 57 U/l (reference up to 35 U/l) was detected. Immunotherapy was paused and MMF switched to CsA. A steady normalization of liver values was obtained after six weeks and therapy with pembrolizumab was continued. Red arrows mark infusion days

Prior to initiation of immunotherapy, screening laboratories for hepatitis A, B and C, were performed and negative. The patient had no medical history for hepatotoxicity in the past, therefore marginally elevated transaminases were tolerated at start of PD-1 inhibitor therapy. Due to parallel induction of MMF and pembrolizumab, blood levels including liver enzymes were measured more frequently than normal. After one cycle of pembrolizumab, the patient’s liver enzymes started to rise slightly (grade 1 common toxicity criteria of adverse events (CTCAE; version 4.0)). The case was discussed in a multidisciplinary team meeting with gastroenterologists and neurologists, concluding that an immune-related hepatitis would rather be unlikely while treated with MMF suspecting MMF-induced toxic liver damage as the most likely diagnosis. At this time, a liver biopsy was not performed due to asymptomatic grade 1 hepatitis. Since levels of aspartate aminotransferase ​​increased on day 15 and were still elevated on day 21, the second pembrolizumab infusion was paused as a precaution. In addition, MMF was switched to CsA at a dose of 2 mg/kg. Within two weeks, after a slight increase of the liver enzymes, transaminases decreased to initial values (Fig. 1) and pembrolizumab was continued.

**Fig. 2:**
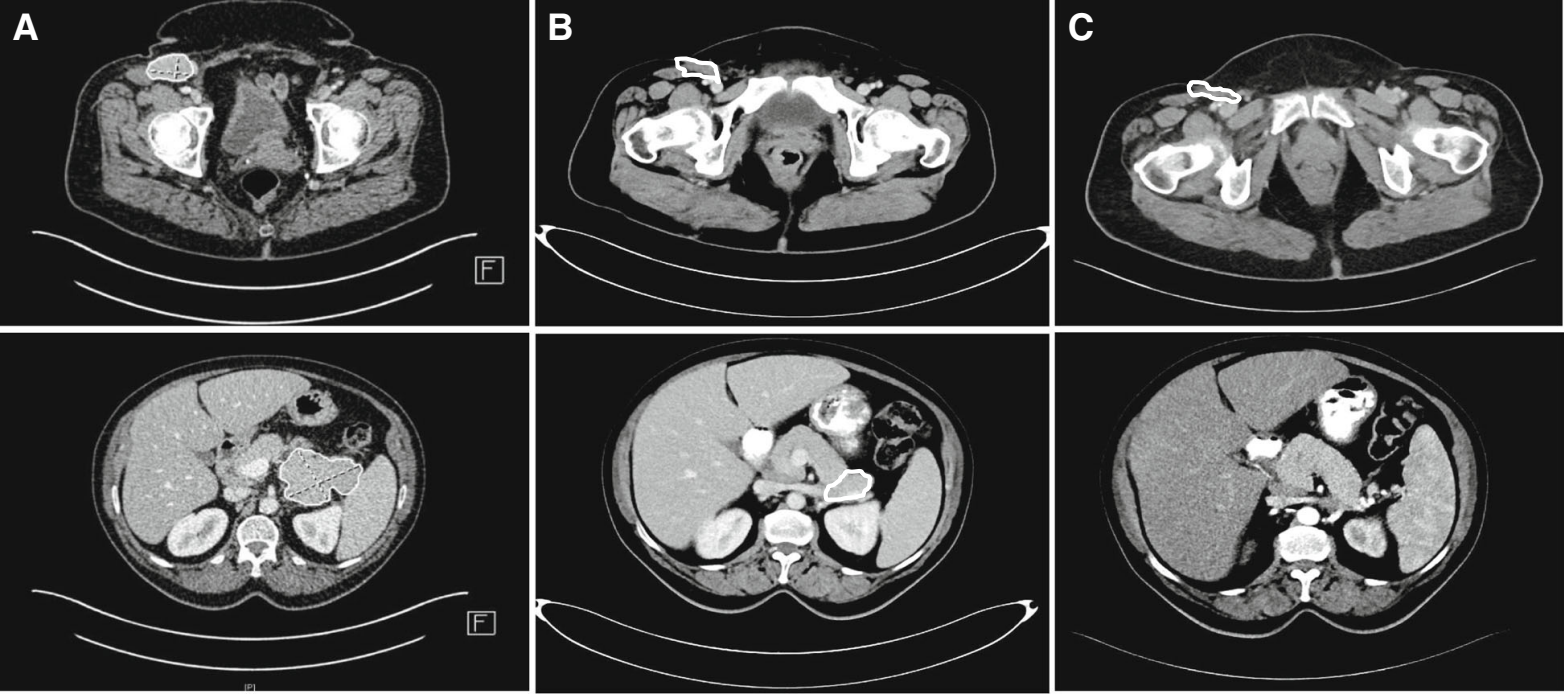
The initial staging from 09/2016 (**a**) shows conglomerated lymph nodes at the inguinal right region and a tumor at the pancreatic tail with an increased glucose metabolism suspicious for tumorous tissue. After six courses of pembrolizumab (**b**) a remission of pancreatic filia and a distinct remission of lymph nodes metastases were detectable. In 02/2018 (**c**) the pre-described tumor at the pancreatic tail is no longer detectable and a constant consolidation at the inguinal right region can be obtained. Furthermore, no new suspicious lesions are present, showing an ongoing partial response to pembrolizumab

Eight weeks after the start of immunotherapy with pembrolizumab, but after only two infusions, the first regular staging with CT scans of the chest, abdomen and brain revealed a mixed response with partial response (PR) of the pancreatic tail tumor and progression of the lymph node metastases of the right groin. As the tumor of the right groin caused severe pain, surgery or localized radiation therapy was recommended. However, after the third course of pembrolizumab the patient reported significant decrease of pain, clinical examination could confirm dramatic regression of the inguinal lymph nodes. Therefore, immunotherapy continued without any further local treatment to the right groin. The patient was in constant surveillance by neurology specialists using the quantitative myasthenia gravis (QMG) score incl. measurement of vital capacity every 3 months and did not experience an exacerbation of MG at any time. She remained on treatment with CsA 125 mg/day and pyridostigmine 60 mg three times daily. The second staging after 23 weeks (six doses) showed further remission of the pancreatic metastasis and distinct remission of the lymph node metastases. So far, immunotherapy with pembrolizumab is still ongoing (27 doses) and CT-imaging of the abdomen revealed a persistent tumor regression of the right inguinal lymph node metastases and a no longer detectable metastasis at the pancreatic tail (Fig. 2) for 65 weeks without any flare of the MG.

## Discussion and conclusion

MCC is a highly aggressive skin cancer which is linked to MCPyV or ultraviolet (UV) radiation [4, 5]. Several risk factors have been identified, primarily immunosuppression, higher age, cumulative UV radiation and other cutaneous tumors, including melanoma [5]. Lately, immunotherapy with PD-1/PD-L1 inhibitors demonstrated a promising approach for metastatic MCC with high and durable response rates ranging between 30 to 60% in the second or first line setting, respectively [5]. Notably, chemotherapy, while inducing high response rates, is not improving overall survival since responses are of very short duration [4].

As PD-1 along with CTLA-4 signaling pathways have been shown to play roles in the maintenance of self-tolerance and modulation of immune responses, irAEs are expected to generate due to an imbalance in the response of T cells to antigens presented by normal cells [16]. Therefore, immune checkpoint inhibitors such as PD-1/PD-L1 inhibitors are suspected to induce irAEs due to impaired self-tolerance from loss of T-cell inhibition [8–10].

In general, it can be distinguished between de novo induced neurological AEs and flares of preexisting neurological disorders by checkpoint inhibition [2, 3]. As patients with preexisting autoimmune disorders are generally excluded from clinical trials with immunotherapeutic agents, safety and efficacy data of immunotherapy in these patients is very limited and mostly of retrospective nature. In two retrospective studies including seven patients with advanced melanoma and preexisting neurological disorders (including one patient with MG, three with Guillain-Barré-syndrome, one with multiple sclerosis, one with chronic inflammatory demyelinating polyneuropathy, one with Bell’s palsy) no flare occurred [2, 3]. Currently no data exist regarding the frequency of exacerbations of preexisting MG in patients who have been treated with checkpoint inhibitors. All previously published case reports reported exacerbations of preexisting MG in patients treated with PD-1 inhibitors [1, 17, 18]. For instance, Makarious et al. reviewed existing literature on immune checkpoint inhibitor-associated MG gathering a total of 23 cases after PD-1 inhibitor, CTLA-4 inhibitor or combination therapy [1]. These patients were further divided into 72.7% (17/23) with de novo MG, 18.2% (4/23) with exacerbations of preexisting MG, and 9.1% (2/23) with exacerbations of subclinical MG^1^. The mean time to onset of MG-symptoms for both de novo presentations and exacerbations was 6.95 (range 2–12) weeks after induction of pembrolizumab, 5.14 (range 2–9) weeks after nivolumab, and 4.75 weeks (range 3–6) after ipilimumab, underlining the early phase of immunotherapy as the most critical [1]. Four out of 13 patients suffering from PD-1inhibitor-induced de novo or subclinical MG died (30.8%), while one MG-induced death was reported in the four patients who showed exacerbations of their preexisting MG after treatment with PD-1 inhibitors (25%) [1]. Compared to this, 2/2 patients treated with the combination of PD-1/PD-L1 inhibitors and CTLA-4 inhibitors died, one due to de novo immune checkpoint inhibitor induced MG, one with unknown status. These cases show that MG-specific-related mortality was comparable between de novo or subclinical MG (30.8%) and exacerbations of preexisting MG (25%) after PD-1 inhibitor treatment [1]. Nevertheless, patient numbers suffering from MG, either de novo or preexisting, are small and further investigations needed to clarify risks and benefits of this patient subgroup.

Taken together, de novo or exacerbations of preexisting MG can be potentially life-threatening and should be monitored in close collaboration with neuromuscular specialists using the QMG score, incl. measurement of vital capacity. In a stable situation, we recommend a QMG scoring every three months, if symptoms get worse, clinical examination should be performed every four weeks. In case of rapid worsening of symptoms, a hospitalization should be considered. Electrophysiological measurements or antibody titers do not necessarily correlate with the clinical severity of MG, therefore repetitive measurement of titers is not helpful.

To our knowledge, currently no data exist regarding reliable factors predicting the risk of checkpoint inhibitor-treated patients with underlying autoimmune diseases and there are minimal data regarding factors predisposing to a flare of the underlying autoimmune disorder. Menzies et al. reported that flares occurred more often in patients with active symptoms, in those requiring immunosuppression when treated with PD-1 inhibitors, and in patients with preexisting rheumatologic disorders [2]. However, patient numbers are small and results should be interpreted with caution. Furthermore, the impact of preexisting immunosuppression on the outcome of immunotherapy remains to be investigated, as so far no prospective studies testing immunosuppressive strategies have been conducted to answer this question. Nonetheless, retrospective studies have shown that patients with immunosuppressants (e.g. steroids) at start of PD-1 treatment had a lower response rate, [2, 19] progression-free survival, and overall survival [19] compared to those not on immunosuppressants.

Our case demonstrates that preexisting autoimmune disorders should not be considered a general contraindication for immunotherapy with PD-1 inhibitors in patients with life-threatening metastatic cancer. PD-1 inhibitor treatment was the favorable choice for our patient, particularly in view of the patients’ progressive, metastatic MCC and the known excellent response rates to PD-1/PD-L1 inhibitors [6, 7]. Nevertheless, selection of therapy and patients should consider the kind and activity of underlying autoimmune disease, as well as tumor entity and its response to PD-1/PD-L1 inhibitors compared to standard of care treatments. It should be kept in mind that the risks/benefit ratio may be less favorable for tumors with relatively low response rates to immunotherapy [20, 21]. The risks and potential benefits of checkpoint inhibitor treatment as well as other therapeutic concepts should be discussed thoroughly in a multidisciplinary setting and require careful and extensive education of the patient and the patient’s family. Exacerbation of underlying MG can be potentially life-threatening and should be monitored in close collaboration with neuromuscular specialists.

## Data Availability

The datasets used and/or analysed during the current study are available from the corresponding author on reasonable request.

## Abbreviations

ACh-R: anti-acetylcholine receptor
AEs: adverse events
CsA: Cyclosporine A
CT: computer tomography
CTCAE: common toxicity criteria of adverse events
CTLA-4: cytotoxic T lymphocyte associated protein 4
ir: immune-related
MCC: Merkel cell carcinoma
MCPyV: Merkel cell polyomavirus
MG: myasthenia gravis
MMF: mycophenolatmofetil
PD-1: programmed cell death protein 1
PD-L1: programmed cell death protein ligand 1
PET: positron emission tomography
PNS: peripheral nervous system
PR: partial response
QMG: quantitative myasthenia gravis
UV: ultraviolet

## References

[1] Makarious D, Horwood K, Coward JIG (2017). Myasthenia gravis: an emerging toxicity of immune checkpoint inhibitors. Eur J Cancer.

[2] Menzies AM, Johnson DB, Ramanujam S (2017). Anti PD therapy in patients with advanced melanoma and preexisting autoimmune disorders or major toxicity with ipilimumab. Ann Oncol.

[3] Gutzmer R, Koop A, Meier F (2017). Programmed cell death protein-1 (PD-1) inhibitor therapy in patients with advanced melanoma and preexisting autoimmunity or ipilimumab-triggered autoimmunity. Eur J Cancer.

[4] Iyer JG, Blom A, Doumani R (2016). Response rates and durability of chemotherapy among 62 patients with metastatic Merkel cell carcinoma. Cancer Med.

[5] Becker JC, Stang A, Nghiem P (2017). Merkel cell carcinoma. Nat Rev Dis Primers.

[6] Nghiem PT, Bhatia S, Lipson EJ (2016). PD-1 blockade with Pembrolizumab in advanced Merkel-cell carcinoma. N Engl J Med.

[7] Kaufman HL, Russell J, Hamid O (2016). Avelumab in patients with chemotherapy-refractory metastatic Merkel cell carcinoma: a multicentre, single-group, open-label, phase 2 trial. Lancet Oncol.

[8] Zimmer L, Goldinger SM, Hofmann L (2016). Neurological, respiratory, musculoskeletal, cardiac and ocular side-effects of anti-PD-1 therapy. Eur J Cancer.

[9] Touat M, Talmasov D, Psimaras D (2017). Neurological toxicities associated with immune-checkpoint inhibitors. Curr Opin Neurol.

[10] Cuzzubbo S, Javeri F, Tissier M (2017). Neurological adverse events associated with immune checkpoint inhibitors: review of the literature. Eur J Cancer.

[11] Johnson DB, Sullivan RJ, Clark JI (2016). Ipilimumab therapy in patients with advanced melanoma and preexisting autoimmune disorders. JAMA Oncol.

[12] Pedersen EG, Pottegård A, Gaist D (2014). Risk of non-melanoma skin cancer in myasthenia patients treated with azathioprine. Eur J Neurol.

[13] Vos M, Plasmeijer EI, Rácz E (2018). Azathioprine to mycophenolate mofetil transition and risk of squamous cell carcinoma after lung transplantation. J Heart Lung Transplant.

[14] Pedersen EG, Pottegård A, Gaist D (2013). Use of azathioprine for non-thymoma myasthenia and risk of cancer: a nationwide case-control study in Denmark. Eur J Neurol.

[15] Perez HC, Benavides X, Lozano E (2017). Basic aspects of the pathogenesis and prevention of non-melanoma skin cancer in solid organ transplant recipients: a review. Int J Dermatol.

[16] Pardoll DM (2012). The blockade of immune checkpoints in cancer immunotherapy. Nat Rev Cancer.

[17] Earl DE, Loochtan AI, Bedlack RS (2018). Refractory myasthenia gravis exacerbation triggered By pembrolizumab. Muscle Nerve.

[18] Cooper DS, Meriggioli MN, Malik R (2017). Severe Exacerbation of Myasthenia Gravis Associated with Checkpoint Inhibitor Immunotherapy. J Neuromuscul Dis.

[19] Arbour KC, Mezquita L, Hellmann MD (2018). Impact of baseline steroids on efficacy of programmed cell Death-1 and programmed death-ligand 1 blockade in patients with non–small-cell lung Cancer. J Clin Oncol.

[20] Forster MD, Devlin MJ (2018). Immune checkpoint inhibition in head and neck Cancer. Front Oncol.

[21] El-Khoueiry AB, Sangro B, Melero I (2017). Nivolumab in patients with advanced hepatocellular carcinoma (CheckMate 040): an open-label, non-comparative, phase 1/2 dose escalation and expansion trial. Lancet.

